# Neutrophils infiltrate into the spiral ligament but not the stria vascularis in the cochlea during lipopolysaccharide-induced inflammation

**DOI:** 10.7150/thno.49121

**Published:** 2021-01-01

**Authors:** Seong Hoon Bae, Jee Eun Yoo, Young Ho Choe, Sang Hyun Kwak, Jae Young Choi, Jinsei Jung, Young-Min Hyun

**Affiliations:** 1 Department of Otorhinolaryngology, Yonsei University College of Medicine, Seoul, Republic of Korea.; 2 Department of Anatomy, Yonsei University College of Medicine, Seoul, Republic of Korea.; 3 Graduate School of Medical Science, Brain Korea 21 Project, Yonsei University College of Medicine, Seoul, Republic of Korea.

**Keywords:** two-photon intravital imaging, neutrophil, cochlea, spiral ligament, stria vascularis

## Abstract

It has been challenging to apply intravital imaging for monitoring the inner ear, as the anatomical location and intricate structure hamper the access of imaging instruments to the inner ear of live mice. By employing intravital imaging of the cochlea in live mice with two-photon microscopy, we investigated neutrophil infiltration into the cochlea tissue and its characteristics under a lipopolysaccharide (LPS)-induced inflammatory state.

**Methods:** Cochlea inflammation was induced by LPS injection to the middle ear. Using two-photon intravital microscopy with specifically designed surgical exteriorization of the cochlea in live mice, we investigated the dynamic features of neutrophils in the lateral wall of the cochlea. The molecular expression pattern of the cochlea lateral wall was also investigated during the LPS-induce inflammation.

**Results:** Despite the contention of whether neutrophils are recruited to the spiral ligament (SL) during inflammation, we observed that LPS-induced inflammation of the middle ear, which mimics acute otitis media, triggered neutrophil migration to the SL in the lateral wall. Notably, massive neutrophil infiltration to the SL occurred 2 days after LPS inoculation, but there was no neutrophil infiltration into the stria vascularis (SV) region. At 1 day after LPS-induced cochlear inflammation, increased mRNA expression of interleukin-1β, interleukin-6 were identified in both the SL and SV, while the *ICAM-1* mRNA expression increased only in the SL. The differential reactivity of ICAM-1 is likely responsible for the different neutrophil recruitment pattern in the cochlea.

**Conclusion:** Intravital imaging of the cochlea revealed that neutrophil recruitment and infiltration during inflammation are spatially controlled and exclusively observed in the SL but not in the SV and organ of Corti.

## Introduction

Two-photon intravital microscopy (TPIM) is one of the most valuable recent developments in biomedical imaging, owing to its non-invasive approach for monitoring deeper structures of the organs or tissues of live animals 1-7. Hence, it is widely applied for intravital imaging because it confers advantages, such as low tissue damage and high penetrating power, which can be attributed to the lower energy requirements, longer wavelength, and nonlinear excitation of the laser 8. TPIM has been used to investigate the cellular structure of various organs and immune responses in neuroscience, ocular research, hepatology, pulmonary research, and many other biomedical fields 3,9-13.

However, monitoring the inner ear with intravital imaging has been challenging because the cochlea is protected by the temporal bone, facial bone, angle of the mandible, and bullae. Furthermore, the cochlea itself exhibits a bony capsulated structure with internal fluid spaces, which makes it difficult to fenestrate it to observe internal structures as we can with the brain tissue inside the skull 14. Meanwhile, researchers have developed many modalities to closely examine the intricately structured cochlea, including immunohistochemistry with paraffin-embedded or frozen sections. However, it was inevitable that specimen corruption and distortion during delicate procedures, and most importantly, fixation of tissue cannot track the dynamic changes in responses.

The lateral wall of the cochlea includes the stria vascularis (SV) and spiral ligament (SL), which are important structures for the maintenance of inner ear homeostasis and immunity. The SV is a capillary network with tight junctions, which is also known as the “blood-labyrinthine barrier”. It was thought that the inner ear was an immunologically privileged organ, based on the tight junctions in the lateral wall of the cochlea, including the SV. Recent studies have revealed that numerous resident macrophages reside in the lateral wall of the cochlea and play an important role in controlling the permeability of the vascular network and immune response following damage such as infection and excessive noise exposure 15,16. It has been reported that leukocytes in the lateral wall of the cochlea mostly comprise macrophages expressing F4/80; the number of macrophages increases during the inflammatory process via the recruitment of monocytes from the blood vessel 16-20.

Moreover, it was thought that neutrophils could not be recruited in the SL during acute inflammation 18,20-22. Indeed, no previous studies have demonstrated this, regardless of the type of damage to the inner ear. However, several studies have identified infiltrated neutrophils in the fluid spaces of the inner ear after a bacterial infection or following direct endotoxin injection 23,24. Hence, it is still unclear whether neutrophils infiltrate the inner ear tissue. Given that excessive infiltration of neutrophils in organs, such as the lungs and heart, may lead to the destruction of the anatomical structure and impair physiological function, it is important to elucidate whether neutrophils infiltrate the cochlea under specific conditions 25,26. From a clinical perspective, this can also elucidate the pathomechanism of sensorineural hearing loss in patients with otitis media.

To explore the response of immune cells in the cochlea, it is necessary to capture the motility and morphology of live immune cells in the well-preserved 3D structure. However, conventional experiments using immunohistochemistry after tissue fixation are not suitable for tracking the movement of immune cells in the cochlea. Therefore, we first aimed to investigate neutrophils in the lateral wall of the cochlea under inflammation induced *in vivo* in response to stimuli via middle ear lipopolysaccharide (LPS) inoculation. Thereafter, we used TPIM to investigate the intravascular and interstitial migration trajectories of neutrophils in live mice. Furthermore, we analyzed the spatial distribution of the recruited neutrophils and explored the molecular mechanism underlying the recruitment. To the best of our knowledge, this is the first study that applied a two-photon microscope for the intravital imaging of mouse cochlea cellular structure. We expect that this study may pioneer a new technique for investigating the actual immune response in the cochlea during inflammation.

## Methods

### Two-photon intravital microscopy

A two-photon microscope LSM7MP (Carl-Zeiss, Oberkochen, Germany) was used to generate the imaging data. Zen 2012 blue edition software (Carl-Zeiss) was used for image acquisition and basic image analysis. Mai-Tai HP Ti:Sa Deep See laser system (Spectra-Physics, Mountain View, CA, USA) was used to generate an excitation laser (wavelengths of 880 nm, 810 nm, and 800 nm for intravital imaging with GFP/Texas red-dextran, three-dimensional cochlea imaging, and intravital imaging with FITC-dextran, respectively). The images were acquired at a resolution of 512 × 512 pixels using band-pass filters with wavelengths in the range of 420-480 nm (blue), 500-550 nm (green), and 575-610 nm (red). We used a 20× objective lens for all experiments.

### Animals

Wild-type C57BL/6 mice were purchased from Orient Bio (Seongnam, Korea). LysM^+/GFP^ mice were produced by interbreeding between wild-type and LysM^GFP/GFP^ mice 27 and were used to visualize immune cells. The mice were maintained in a specific pathogen-free environment in the animal facility at the Avison Biomedical Research Center in Yonsei University College of Medicine, Seoul, Korea. Four to eight-week-old male and female mice were used in the experiments. All animals were carefully maintained in cages without exposure to noise. The animal experiments were approved by the Institutional Animal Care and Use Committee at the Yonsei University College of Medicine (Protocol number 2019-0182).

### LPS stimulation of the cochlea

A total of 5 µL of LPS (Sigma Aldrich, St. Louis, MO, USA) solution (5 mg/mL or 1.25 mg/mL) was injected into the middle ear cavity of the mouse through the tympanic membrane under general anesthesia induced with zoletil (Virbac, Carros, France) (30 mg/kg) and xylazine (Bayer, Leverkusen, Germany) (10 mg/kg). As the round window of the cochlea is located at the posterior tympanic cavity, the tympanic injection was administered consistently at the site of the posterior tympanic membrane under the light microscope. LPS diffuses into the cochlea via the round window membrane 28. For *in vivo* blockade of integrin LFA-1 and Mac-1, blocking antibodies (BioLegend, San Diego, CA, USA), namely, anti-M17/4 and anti-M1/70, respectively, were injected (50 μg/animal) into the retro-orbital sinus under general anesthesia one day after LPS inoculation into the middle ear.

### Cochlea exteriorization for intravital imaging

**Figure 1:**
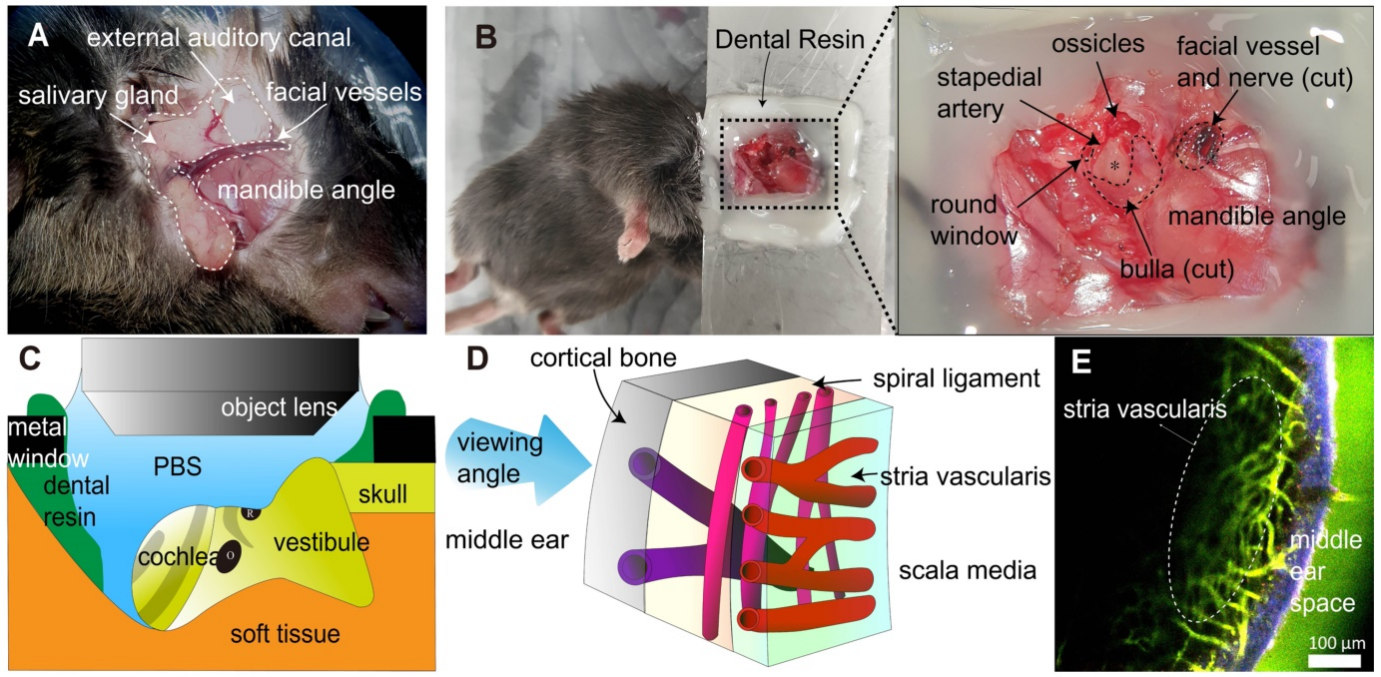
** Surgical exteriorization for the intravital imaging of mouse cochlear lateral wall.** (A) The anatomical remarks in the subcutaneous tissue to access cochlea were identified after posteroinferior auricular skin flap elevation. (B) The anatomical remarks related to the cochlea (asterisk). The dental resin was applied between tissue and metal window to seal liquid media. (C) Schematic diagram of the intravital imaging set up. The objective lens of two-photon microscopy approaching the mouse cochlea. The round window of the cochlea is at the top. Dental resin sealed the PBS medium. The gray strip indicates the stria vascularis. O, oval window. R, round window. (D) Schematic diagram of the cochlear lateral wall anatomy shows three different vessels layer by layer, which demonstrate different running directions. (E) Intravital image of the cochlear lateral wall, showing stria vascularis capillary (marked) and spiral ligament vessel, which run perpendicular to each other. The image was obtained after i.v. injection of FITC-dextran. The area in *blue* shows the second harmonic generation of cortical bone, whereas the area in *green* shows blood vessel image enhanced by FITC-dextran.

Mice were i.p. injected with zoletil (30 mg/kg) and xylazine (10 mg/kg) to induce general anesthesia. This was followed by surgical procedures. A posteroinferior auricular skin incision was made, and the subcutaneous tissue was dissected until the masseter muscle on the angle of the mandible was exposed (Figure 1A). The facial vessel under the salivary gland was blocked by electrocautery to prevent severe bleeding. The facial nerve was identified beneath the external auditory canal (EAC) and cut. The EAC was severed circumferentially, and the tympanic membrane was exposed. The bulla and tympanic membrane were carefully removed to expose the cochlea. After surgery, the mice were placed in the lateral decubitus position, and their head was firmly attached to the metal window with dental resin. Notably, the mouse head should be properly positioned such that the round window is located at the top for easy access to the objective lens (Figure 1B-C). As this study used a water immersion objective lens, it was important to cover the imaging area with water or phosphate buffered saline (PBS) during intravital imaging. Therefore, dental resin was applied to create a dam-like barrier to prevent PBS leakage. After the cochlea was exposed by surgery, 200 μL of dye-conjugated dextran (500 μg/animal) was injected into the retro-orbital sinus.

### Two-photon intravital imaging

To investigate intravascular crawling and interstitial migration, 50-58 sequential planes were acquired in the *z* dimension (1 µm each) to form a *z* stack. The pixel dwelling time was 1.58 µs, and the acquisition cycle was defined as 60 s. To investigate rolling and adherent cells, a single plane image was acquired with a pixel dwelling time of 1.58 µs and an acquisition cycle of 1 s. The mice were placed on the warming pad (37 °C) during intravital imaging. With each animal, two sessions of intravital imaging were performed for 20-40 min of each session. Intravital imaging experiments were performed with the untreated mice, 1 day after PBS treatment for the control group. After LPS inoculation, the intravital imaging experiments were performed at 6 h, 1 day, 2 days, and 4 days. All experiments were independently repeated at least three times in each experimental group. All animals survived after intravital imaging and were later euthanized.

### Image analysis

For intravital neutrophil analysis, GFP-positive cells in an area of 50,000 μm^2^ including the SV were analyzed for 20 min to investigate the intravascular crawling and interstitial migration and 10 min for investigating rolling and adherence. In rolling and adherent cell analysis, cells moving at a rolling velocity of < 30 µm/s over 5-30 s were defined as rolling cells, and cells that remained stationary for more than 30 s were defined as adherent cells 29,30. In migrating cell analysis, the intravascular crawling cells were defined as cells that appeared in more than four consecutive sequences (3 min), and the interstitial migrating cells were defined as mobile cells located outside of the vessels. Cell tracking was created using ImageJ (NIH). The track analysis was manually performed using IMARIS (Bitplane) and PRISM (GraphPad). The track speed was defined as the length of track per duration, and the displacement was defined as the straight distance of cell location from the first image to the last image. The meandering index was calculated as the displacement of track divided by the length of the track. The duration was calculated as the consecutive time that cells met the criteria for rolling/adherent cells.

### Three-dimensional cochlea imaging

Mice that were used in the intravital imaging were euthanized in a CO_2_ chamber, and the cochlea was collected from the contralateral temporal bone. Each obtained cochlea was fixed in 4% paraformaldehyde overnight at 4 °C on a rotating plate (30 rpm). For decalcification, the fixed temporal bone was incubated overnight in a 1:3 ethylenediaminetetraacetic acid (EDTA)/PBS solution at 4 °C on a rotating plate (30 rpm). After decalcification, the temporal bone was washed in PBS for more than 2 h at room temperature on a rotating plate (105 rpm). Decalcified temporal bone was placed on the dish with a silicon bond and immersed in PBS before imaging 31. The collected images were reconstructed using Zen software (Carl-Zeiss). Neutrophils were quantified after sagittal reconstruction with a 10 μm depth. The SV and adjacent SL were compared (~6000 μm^2^). Three random sagittal images per cochlea were selected, and three cochleae per group were analyzed.

### Immunohistochemistry

The isolated cochleae were obtained by microdissection. Tissues were fixed by submerging in 4% formaldehyde at 4 °C for 24 h. After washing twice with PBS, the fixed temporal bones were decalcified for 24 h in 10% EDTA/PBS. The whole-mounted tissues were blocked with 10% donkey serum and incubated with target-specific primary and secondary antibodies at 4 °C overnight. The samples were then mounted with a mounting solution (Sigma-Aldrich) and viewed under an LSM780 confocal microscope (Carl-Zeiss). For paraffin sectioning, the tissues were embedded in paraffin for standard histological examination. The paraffin blocks were sliced into 5 mm-thick sections in the mid-modiolar plane using a microtome (Leica Biosystems, Nussloch, Germany). Deparaffinization was performed on the sections as well as the whole-mounted tissues with a series of washes with xylene, ethanol, and PBS. After incubation in Tris-sodium citrate at 95 °C for antigen retrieval, the tissues were blocked with 10% donkey serum and incubated with target-specific primary and secondary antibodies at 4 °C overnight.

### Western blotting

The heads of mice were separated from the body immediately after sacrifice. The lateral wall (consisting of the SV and SL) of the cochlea was carefully dissected in ice-cold PBS. The SV and SL were manually separated under a light microscope. Six cochleae from three mice were pooled for each group. The SV and SL were ground and lysed in sodium dodecyl sulfate (SDS) lysis buffer. Lysed samples were mixed with sample buffer, separated by SDS-polyacrylamide gel electrophoresis, and transferred to a nitrocellulose membrane. The membrane was incubated with appropriate primary and secondary antibodies. Protein bands were detected by enhanced chemiluminescence (Amersham Bioscience, Little Chalfont, UK). The intensity of bands from three blots (nine biological replicates) were quantified using ImageJ (NIH).

### Quantitative real-time polymerase chain reaction (qPCR)

The heads of mice were separated from the body immediately after sacrifice. The lateral wall (consisting of the SV and SL) of the cochlea was carefully dissected in RNAlater solution (Thermo Fisher, Waltham, MA, USA). The SV and SL were manually separated under a light microscope. Six cochleae from three mice were pooled per group. The SV and SL were ground and lysed using TRIzol (Thermo Fisher). Purification of mRNA was performed using PureLink RNA mini kit (Ambion, Austin, TX, USA) according to the manufacturer's instructions. Purified RNA samples were reverse-transcribed using an AccuPower CycleScript RT PreMix (dT20) kit (Bioneer, Daejon, Korea). Amplification was performed using a Quantstudio 3 real-time PCR system (Applied Biosystems, Foster City, CA, USA) using PowerUp^TM^ SYBR Green Master Mix (Applied Biosystems).

### Flow cytometry

After both sides of the temporal bones were isolated from the mice, the bony capsule of the cochlea was carefully removed in PBS. The whole cochlea tissue was extracted from the cochlear bony capsule and temporal bone. After the cochlear tissue was transferred to a new dish, the tissue was trypsinized for 10 min, followed by gentle grinding on a 40 μm filter. The cells were stained with dye-conjugated anti-CD11b antibody (Biolegend, 1:200) and anti-Ly6G antibody (Biolegend, 1:200) for 30 min at 4 °C. Flow cytometry was performed using BD LSR II (Becton Dickinson, Franklin Lakes, NJ, USA). The analysis was performed using the FlowJo software (Becton Dickinson).

### Reagents and resources

Texas-Red dextran (70 kDa, Ex/Em 595/615 nm, Thermo Fisher) and FITC dextran (70 kDa, Ex/Em 494/521, Thermo Fisher) were used to visualize vessels in the intravital image. LPS (5 mg/mL, Sigma Aldrich) was used and diluted as necessary. PE-conjugated anti-Ly6G antibody (clone 1A8, 127608, BioLegend) and FITC-conjugated anti-Ly6G antibody (clone 1A8, 127606, BioLegend) were used to stain neutrophils. APC-conjugated anti-F4/80 antibody (clone BM8, 17-4801-82, eBioscience) and PE-conjugated anti-F4/80 antibody (clone BM8, 12-4801-82, eBioscience) were used to stain the macrophages. APC-conjugated anti-CD11b antibody (clone M1/70, 101212, BioLegend) and FITC-conjugated CD11b antibody (M1/70, 101206, BioLegend) were used for gating myeloid cells in flow cytometry. Antibodies against LFA-1 (clone M17/4, 101118, BioLegend) and Mac-1 (clone M1/70, 101248, BioLegend) were used for *in vivo* blocking experiments. Anti-ICAM-1 antibody (ab222736, Abcam, Cambridge, UK) was used for western blotting. The following primer sequences were used in PCR: *PECAM-1* (F: AACAGAAACCCGTGGAGATG, R: GTCTCTGTGGCTCTCGTTCC), *ICAM-1* (F: ATTCGTTTCCGGAGAGTGTG, R: CAGCACCGTGAATGTGATCT), *GAPDH* (F: AGAACATCATCCCTGCATCC, R: CACATTGGGGGTAGGAACAC), interleukin-1β (*IL-1β*) (F: CCCTGCAGCTGGAGAGTGTGGA, R: TGTGCTCTGCTTGTGAGGTGCTG), interleukin-6 (*IL-6*) (F: CCTCTCTGCAAGAGACTTCCAT, R: AGTCTCCTCTCCGGACTTGT), claudin-11 (*CLDN11*) (F: GACCTGCAGCTACACCATC, R: GGGGTTTGCAGTGGTAGAGA), and occludin (*OCLN*) (F: ACCCGAAGAAAGATGGATCG, R: CATAGTCAGATGGGGGTGGA).

### Statistical analysis

Continuous values are described as the mean ± SEM. A nonparametric comparison test (Mann-Whitney test) was used to compare two independent groups. Analysis of variance (ANOVA) was used to compare more than three independent groups. A two-tailed *P*-value < 0.05 was considered statistically significant. Statistical analysis was conducted using SPSS software ver. 25 (IBM).

## Results

### Surgical exteriorization of mouse cochlea for two-photon intravital imaging

To access the cochlea, which is deeply located in the head, surgery must be performed under general anesthesia. Therefore, we designed surgical procedures to exteriorize the cochlea of live mice and perform intravital imaging of the cochlea in live mice (Figure 1A-B). Blood vessels in the cochlear lateral wall were visualized with the i.v. injection of FITC-dextran or Texas Red-dextran via TPIM (excitation laser wavelength: 800 nm and 880 nm, respectively). Three types of vessels were identified, and each type had a different depth of location and running direction (Figure 1D-E). The cortical vessel was located the most superficially beneath the cortical bone and radiated randomly. The SL vessel was located between the cochlea bone and SV and radiated along the modiolar axis. The SV capillary was located in the deepest structure in the lateral wall in contact with the scala media, and it radiated along the cochlear turn. The vessels in the SL and SV demonstrated a perpendicular running axis.

### Intravascular migratory pattern of neutrophils in LPS-stimulated cochlea

We investigated the behavior of neutrophils during inflammation using intravital imaging. LysM-GFP^+/-^ mice were used to monitor the motility and morphology of neutrophils in the cochlea. It has been reported that green signal cells of LysM-GFP^+/-^ mice are primarily derived from neutrophils and certain monocytes or macrophages with dim green fluorescence 27,32. Intravital images of LysM-GFP^+/-^ mice demonstrated some green signal cells in the SL. The dendritic morphology and immobility suggested that these cells could be macrophages that commonly reside in the cochlear lateral wall.

**Figure 2:**
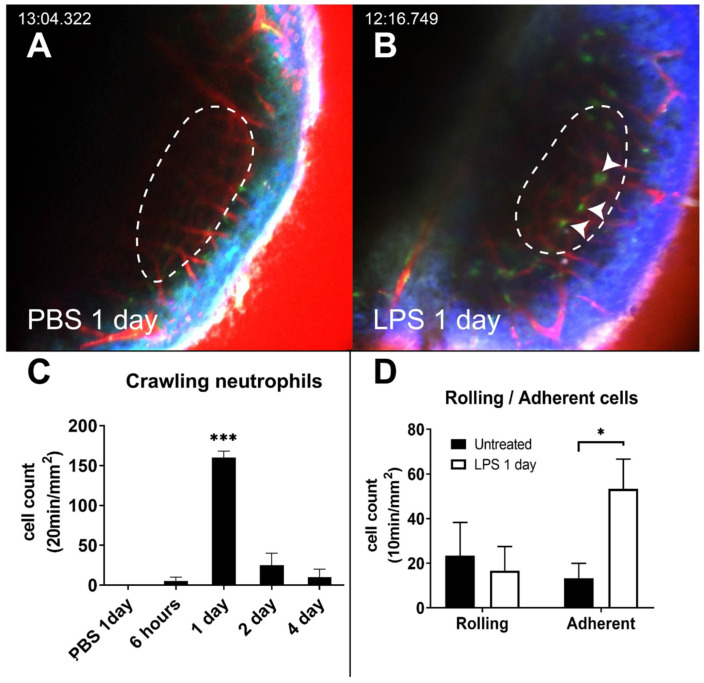
** Intravascular crawling neutrophils in the cochlea vessels 1 day after LPS stimulation.** (A and B) Intravital image of cochlear lateral wall in the LysM-GFP^+/-^ mouse 1 day after the inoculation of the middle ear with PBS (A) and 1 day after the inoculation of the middle ear with LPS (B). The images were obtained after i.v. injection of Texas Red-dextran. The stria vascularis region is marked by the dotted line. *Arrowhead* indicates crawling cells in the vessel. *The green* region shows LysM-GFP positive cells. The *red* region shows blood vessels stained with Texas Red-dextran. The *blue* region shows the second harmonic generation of cortical bone by lasers. Scale bar = 100 µm. (C) Intravascular crawling neutrophil counts from the stria vascularis corresponding to the interval after LPS inoculation. (D) Rolling and adherent neutrophil counts from the stria vascularis were compared between untreated and LPS inoculation. (*Denotes *P*-value < 0.05, *** denotes *P*-value < 0.001 in *post hoc* analysis after ANOVA.)

Under both the PBS-inoculated and untreated conditions, GFP-positive cells were barely observed inside the vessels. This can be attributed to the fact that the cells do not tend to interact with the luminal surface of the endothelium and flow with the blood (Figure 2A and Video S1). Interestingly, the intravascular crawling GFP-positive cells were easily identified 1 day after LPS inoculation (Figure 2B-C and Video S2). In the LPS inoculation group, the number of adherent neutrophils significantly increased. However, the number of rolling cells did not increase compared to that in the untreated group (Figure 2D). The dynamic properties of the rolling cells also did not differ between the two conditions (Figure S1). The number of intravascular crawling cells that were GFP-positive decreased rapidly in 2 days.

### Interstitial migratory pattern of neutrophils in the LPS-stimulated cochlea

**Figure 3:**
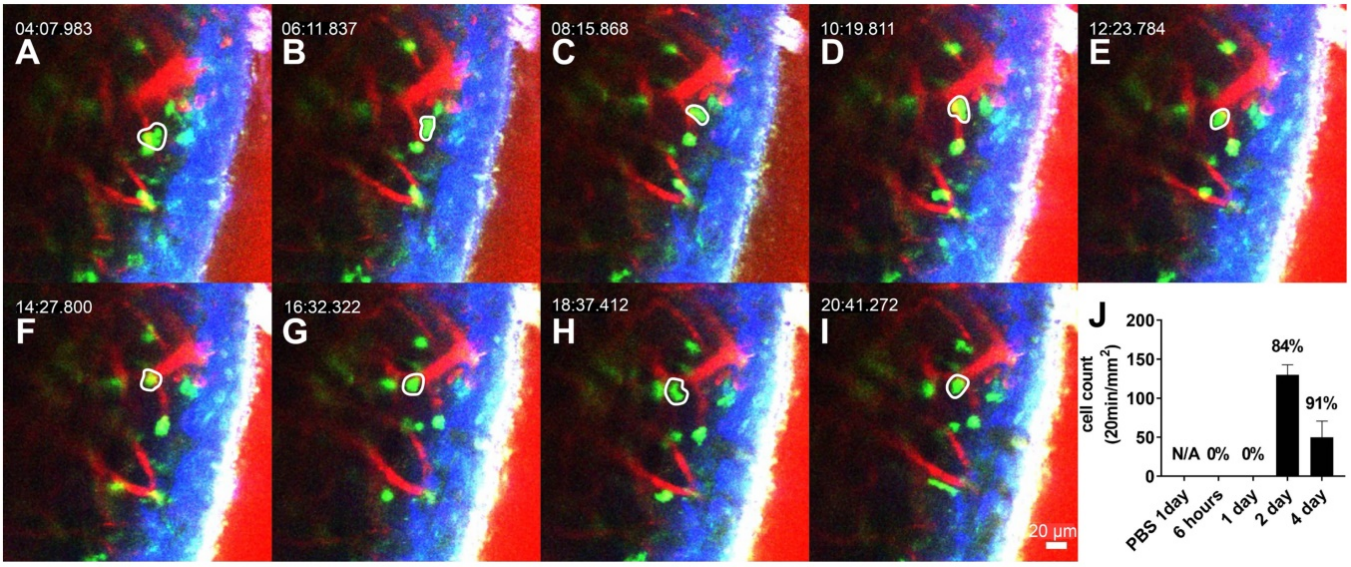
** Interstitial migrating neutrophils in the spiral ligament 2 days after middle ear inoculation with LPS.** (A-I) Intravital image of the cochlear lateral wall in the LysM-GFP^+/-^ mouse shows serial time lapse of a migrating cell (white-lined polygon). The images were obtained after i.v. injection of Texas Red-dextran.* The green* region shows LysM-GFP positive cells. *The red* region shows blood vessels stained with Texas Red-dextran.* Blue* region shows the second harmonic generation of cortical bone by lasers. Scale bar = 20 µm. Time interval = 2 min. (J) Quantification of the interstitial migrating cells. The percentage above the graph indicates interstitial migrating neutrophil fraction calculated as the number of interstitial migrating neutrophils divided by the total number of intravascular crawling neutrophils and interstitial migrating cells.

The interstitial migrating neutrophils were observed 2 days after LPS inoculation to the middle ear. The cells in the interstitial space were easily discernible by the spatial relationship with the blood vessels and were robustly mobile (Figure 3A-I, and Video S3). Intravital images showed that the number of migrating cells peaked 2 days after LPS inoculation and was reduced after 4 days (Figure 3J). Track analysis was performed to confirm the dynamic differences between intravascular crawling and interstitial migration of these cells (Figure S2). First, we observed that the displacement of GFP-tagged neutrophils was significantly different between neutrophils in intravascular crawling and interstitial migration states. The velocity and meandering index of neutrophils were significantly lower in the interstitial migrating cells than in the intravascular crawling cells. The mean velocity of crawling cells and migrating cells were consistent with previous studies that reported the speed of neutrophil crawling and migration *in vivo* 33,34.

### Neutrophils did not infiltrate the SV during inflammation

**Figure 4:**
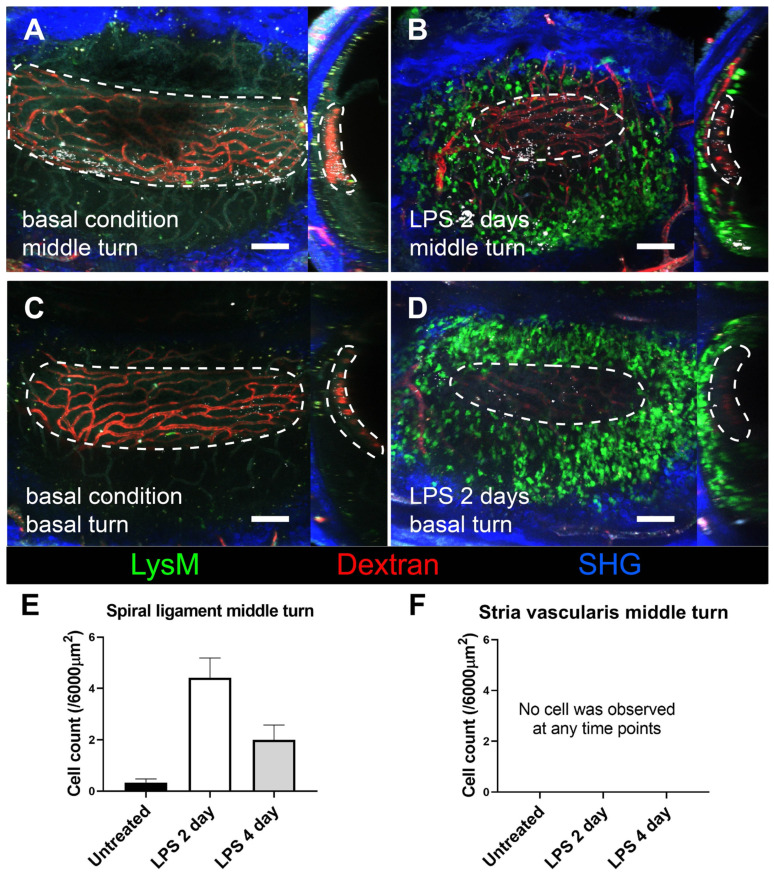
** Stria vascularis as a neutrophil-free region during cochlea inflammation.** A three-dimensional image of cochlear lateral wall using two-photon intravital microscopy shows middle turn of the untreated cochlea (A), middle turn of the inflamed cochlea with middle ear inoculation with LPS 2 days before euthanization (B), basal turn of the untreated cochlea (C), and basal turn of the inflamed cochlea with LPS middle ear inoculation 2 days before euthanization (D). In each cochlea image, the X-Y plane is presented on the left side, and the Y-Z plane of the sagittal reconstructed image is presented on the right side. The region of the stria vascularis is marked with a dotted line. After sagittal reconstruction, neutrophils were quantified in the spiral ligament adjacent to the stria vascularis (~6000 µm^2^) (E) and in the stria vascularis (~6000 µm^2^) (F). Notably, neutrophils were absent in the stria vascularis at any time point. The contralateral cochlea was obtained from the mouse after intravital imaging performed after i.v. injection of Texas Red-dextran. The *green* region shows LysM-GFP positive cells. *The red* region shows blood vessels stained with Texas Red-dextran*.* The *blue* region shows the second harmonic generation of cortical bone by lasers. All images were stacked at 30 µm. Scale bar = 100 µm.

**Figure 5:**
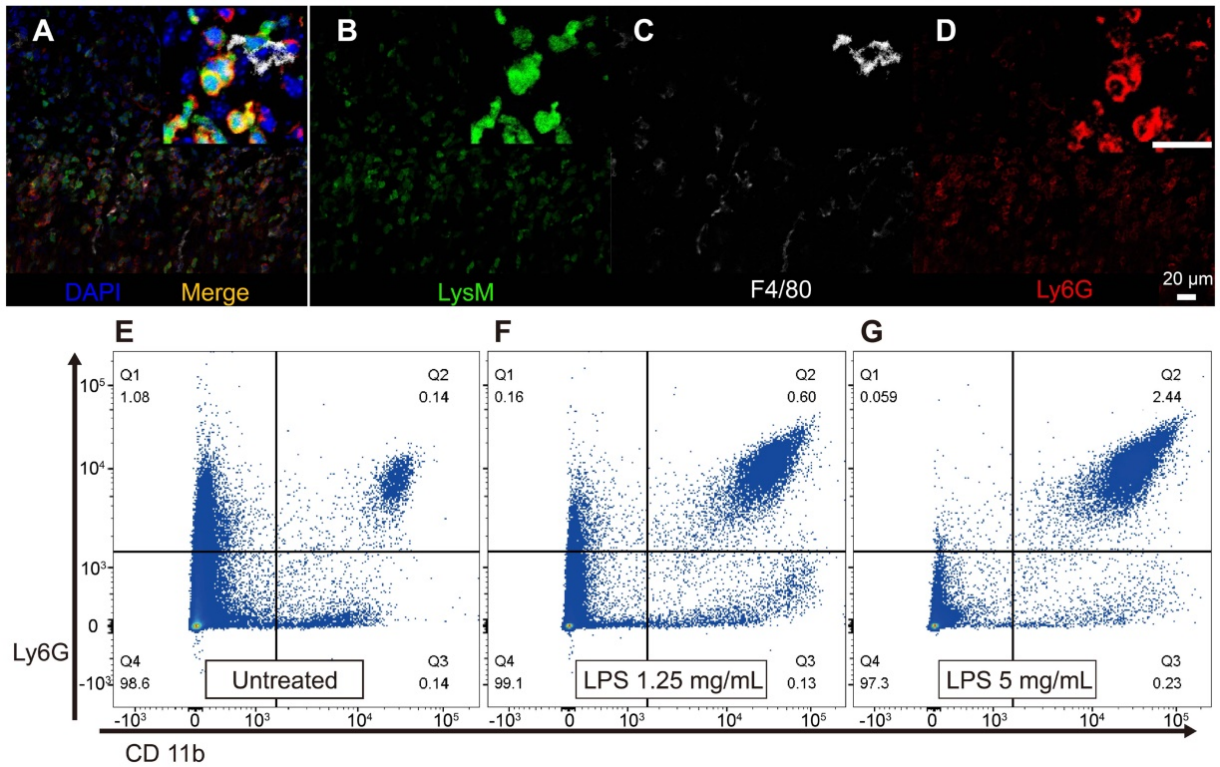
** Infiltrated LysM-GFP positive cells in the cochlear lateral wall 2 days after LPS inoculation are mainly neutrophils and the number of neutrophils increases in proportion to LPS concentration.** Immunohistochemistry was performed with cell-specific markers, and a magnified image is located in the upper right corner of each image. (A-D) The merged image (A) shows LysM-GFP positive cells in green (B), F4/80-positive cells in white (C), and Ly6G-positive cells in red (D). *Blue*, DAPI. (E-G) Flow cytometry was performed with a different LPS dose, such as untreated control (E), LPS concentration of 1.25 mg/mL (F), and LPS concentration of 5 mg/mL (G). The *y*-axis represents the fluorescence intensity of Ly6G, and the *x*-axis represents the fluorescence intensity of CD11b. Cells in the Q2 area (Ly6G+, CD11b+) were defined as neutrophils. Scale bar = 20 µm.

We further investigated how GFP-positive cells are localized based on the three-dimensional (3D) image of the lateral wall in the exteriorized cochlea obtained using two-photon microscopy. Consistent with the other experiments described above, massive neutrophil infiltration was observed in SL 2 days after LPS inoculation. The 3D image showed that neutrophils infiltrated the entire region of the SL, from the basal to the middle turn of the cochlea. Interestingly, neutrophils were absent in the SV at any given interval (Figure 4). A substantial number of the infiltrating GFP-positive cells in the SL observed 2 days after LPS inoculation were identified as neutrophils, which were stained with the neutrophil-specific marker anti-Ly6G antibody (Figure 5A-D). A small number of GFP-positive cells were stained with the antibody against F4/80, a monocyte/macrophage marker. The cells were morphologically different from the cells stained with anti-Ly6G antibody. None of the GFP-positive cells was stained with the anti-Ly6G antibody in the cochlea obtained from the control and PBS-injected mice. The increase in the number of infiltrating neutrophils in the cochlea was also confirmed by flow cytometry. The Ly6G and CD11b double-positive cells indicated that the number of neutrophils increased dose-dependently 2 days after LPS inoculation (Figure 5E-G).

### Different inflammatory responses between the SV and SL

**Figure 6:**
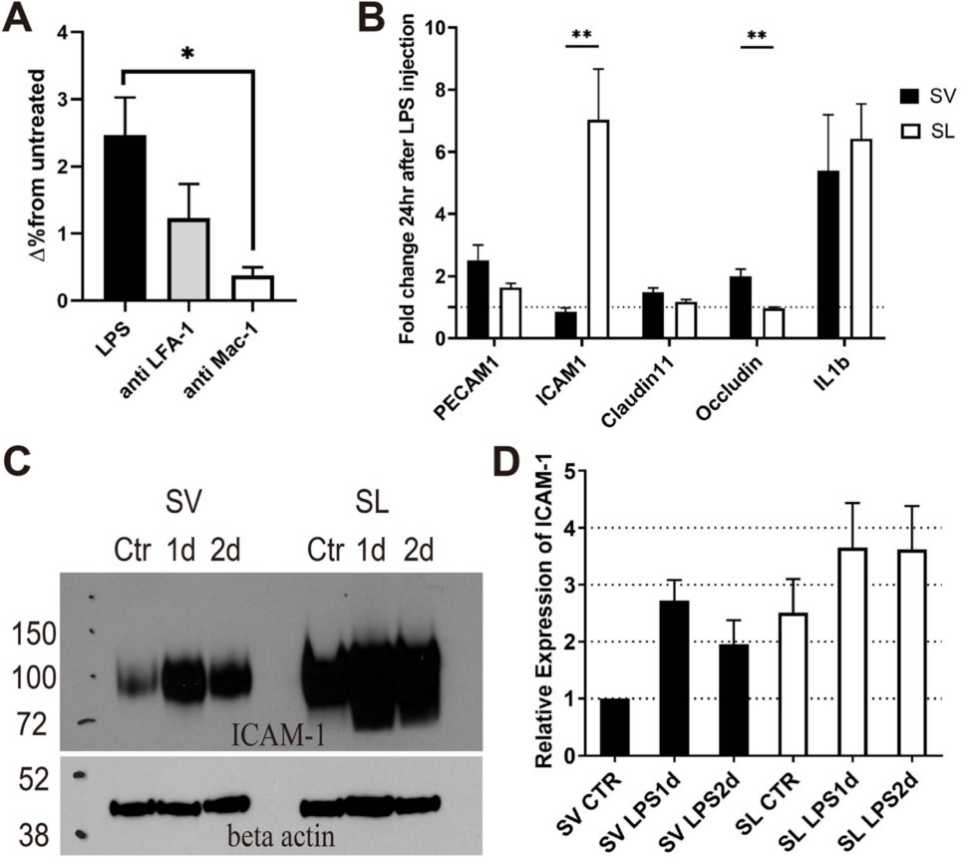
** Neutrophil infiltration depends on ICAM-1 in the cochlea, and ICAM-1 expression is lower in the stria vascularis than in the spiral ligament.** The *in vivo* blockade of the ICAM-1 ligands (LFA-1 and Mac-1) decreases neutrophil infiltration in the cochlea after LPS inoculation. (A) Flow cytometry analysis showed a statistically significant difference in the number of neutrophils (Ly6G+, CD11b+) in the cochlea with LPS without blocking and LPS with anti-Mac-1 antibody treatment *in vivo*. (B) The fold change in mRNA expression at 1 day after LPS injection compared to basal conditions analyzed by qPCR. Notably, *ICAM-1* mRNA expression was not increased in the stria vascularis, although *IL-1β* mRNA expression was increased. (C) Western blot analysis using anti-ICAM-1 antibody. Beta-actin was blotted on the same membrane after stripping. An representative image of Western blot was shown from three independent experiments. Six cochleae from three mice were pooled for each group per experiment. (D) Western blot was quantified by the intensity of ICAM-1 band normalized by the beta-actin band. Relative expression was compared to 'SV Ctr' (untreated group) lane. SL, spiral ligament. SV, stria vascularis (** denotes *P*-value < 0.005, in post-hoc analysis after ANOVA).

Although adherent neutrophils increased in SV vessels after inflammation, interstitially infiltrated neutrophils were not observed in the SV (Figures 2 and 4). To determine the reason why the interstitial migration of neutrophils was demonstrated only in the SL, we first attempted to confirm if the mechanism of neutrophil infiltration in the cochlea also depends on ICAM-1. *In vivo* blocking of LFA-1 and Mac-1, which are the counter ligands for ICAM-1, significantly decreased the number of infiltrated neutrophils 2 days after LPS inoculation (Figure 6A). In particular, Mac-1 was shown to play a predominant role in neutrophil infiltration, which was consistent with previous studies conducted in cremaster venules in response to TNF-α treatment 33,35. We previously reported that LFA-1 and Mac-1 regulate neutrophil migration differently during inflammation 36; therefore, it is also considered that these β_2_ integrins may affect neutrophil migration differently in the cochlea. This result implies that the neutrophil infiltration cascade to the SL also depends on ICAM-1.

Next, we investigated the molecular mechanism limiting neutrophil migration to the SV. Because the SV was easily detachable from the SL under the light microscope, mRNA expression between the two regions could be quantified and compared using qPCR. Compared to basal conditions, the mRNA expression of *ICAM-1*, *IL-1β*, and *IL-6* was markedly increased in the SL 1 day after injecting the middle ear with LPS (Figure 6B and Figure S3). However, in the SV, the mRNA expression of *ICAM-1* rarely changed, although the mRNA expression of *IL-1β* and *IL-6* was increased (Figure 6B and Figure S4). While the mRNA expression of *ICAM-1* showed a significantly higher fold change in the SL than in the SV, the mRNA expression of *OCLN* (associated with tight junction) showed a higher fold change in the SV than in the SL. Western blotting results revealed that ICAM-1 levels increased 1 and 2 days after LPS injection in both the SV and SL (Figure 6C); however, an increase in ICAM-1 levels was observed at all time points in the SL compared to the SV. The increase in ICAM-1 levels in the SV was consistent with neutrophil crawling 1 day following LPS injection (Figure 2A).

## Discussion

The cochlea faces many environmental challenges, such as middle ear bacteria and continuous noxious sound stimulation. Given that the inner ear is easily exposed to bacterial endotoxins from the middle ear mucosa and noxious noise, LPS and noise frequently jeopardize the hearing function of the cochlea 18,20,22,37,38. The SL is the circumferential tissue surrounding and protecting the cochlea from the outside. Recently, we reported that cochlin, the most abundant protein in the SL, demonstrates a protective immune activity against the infiltrating bacteria from the middle ear 39. However, the compartment of the cochlea where infiltrated immune cells are localized and the mechanism through which the structure of blood vessels in the cochlea contributes to immune cell recruitment remain elusive.

In the present study, we observed that the SL was not a neutrophil-free region, based on the massive neutrophil migration in the SL shown 2 days after LPS inoculation into the middle ear. Considering that neutrophils are typically known to be recruited within several hours of stimulation, the immune response lagged relatively as LPS should be diffused into the cochlea via the round window membrane 28.

Another inflammatory trigger of the SL is excessive noise exposure to the cochlea 37,40. In contrast to LPS stimulation, noise exposure to the cochlea may not induce the interstitial migration of neutrophils in the lateral wall, as shown in many previous studies 18,20-22. LPS and excessive noise exposure might have different inflammatory pathways because LPS is recognized as a pathogen-associated molecular pattern, but debris from the degenerated cells after noise exposure is recognized as a damage-associated molecular pattern (DAMP) 41. However, this difference cannot sufficiently explain the disparity in neutrophil recruitment as burn damage, which representatively induces inflammation via DAMP and demonstrates massive neutrophil influx in other organs 42-44. Although noise exposure induces permanent hair cell damage and hearing loss, it might be a much weaker immunological stimulation than LPS inoculation and might not be enough to induce neutrophil infiltration. Another speculation is that the SL in the cochlea avoids massive recruitment of neutrophils even after DAMP activation in response to acoustic damage to prevent subsequent tissue damage due to excessive inflammation. Taken together, cochlear immune responses in the SL are diverse from the viewpoint of neutrophil recruitment depending on the type of immune response triggers. Further research should be conducted to elucidate the mechanism underlying the discrepancy in the immune response.

This study showed that neutrophils interact with the endothelial cells in the vessels of the cochlear lateral wall after LPS challenge. In particular, cells adherent to the vessel walls were more frequently observed in the inflamed SV than in the basal condition, whereas intravascular rolling cells were barely observed in both the basal and inflamed SV (Figure 2). Moreover, rolling patterns, such as velocity, displacement, meandering index, and duration of rolling cells, were not significantly different in the SV before and after LPS stimulation (Figure S1). This phenomenon can be partially explained by very slow blood flow in SV vessels (30-100 μm/s in guinea pig) as leukocyte rolling is critically affected by the shearing force of the flow 29,45. However, because our device for TPIM is not able to obtain more than 1 frame per second, rolling cells faster than 30 μm/s were not included in the analysis and can bias the result.

Meanwhile, the number of crawling cells increased significantly in the SV 1 day after LPS inoculation. However, the number of migrating neutrophils in the interstitium exclusively increased in the SL but not in the SV. Marginal cells in the SV play distinct roles in maintaining the endocochlear potential in the cochlea, which is indispensable for hearing function 46. Thus, it is important to maintain immune privilege in the SV despite immunological challenges. For maintaining continuous and stable high potassium levels in the scala media, the cellular alignment of the SV should always be secured. The results of the present study indicated that ICAM-1 expression markedly differed between the SV and SL after LPS challenge. ICAM-1 plays a critical role in the adhesion, crawling, and transendothelial migrating phase of leukocytes. ICAM-1 protein level and neutrophil crawling peaked 1 day after the LPS challenge in the SV, but the mRNA expression of *ICAM-1* did not increase at that time point. The peak of *ICAM-1* mRNA expression in the SV might be earlier than 1 day after LPS injection, given that endotoxins injected into the middle ear are detected in the inner ear approximately 12 h after injection 47. Additionally, increased ICAM-1 protein level in the SV did not seem to be sustained until day 2 compared to that in the SL. In contrast, *ICAM-1* mRNA expression in the SL was distinctively increased at 1 day after LPS injection. ICAM-1 protein level was also higher in the SL than in the SV at basal conditions and 1 day after LPS injection. Furthermore, the increased ICAM-1 protein level was relatively sustained in the SL compared to the SV at 2 days after LPS injection. Nevertheless, care should be taken to interpret this result because ICAM-1 can be expressed in other cells as well as endothelial cells. However, it is challenging to isolate capillaries from the tiny tissue without damaging them. Furthermore, *PECAM-1, CLDN11, IL-1β, and IL-6* gene expression after LPS challenge were not significantly different between SV and SL, contrary to *ICAM-1*. Taken together, the different responses of ICAM-1 expression likely be a cause of limited neutrophil interstitial migration in the SV. However, the exact molecular pattern specifically in the endothelial cells of SV and SL should be clarified in future studies.

In addition, abundant tight junctions, including claudins, and lack of extracellular matrix in the SV may act as a barrier against immune cell infiltration 48-50. Under basal conditions, the SV showed higher mRNA expression of *CLDN11* and *OCLN*, which are tight junction-associated genes (Figure S4). In addition, the fold change in *OCLN* mRNA expression was significantly higher in the SV than in the SL at 1 day after LPS injection (Figure 6B). Histological analysis using Masson's trichrome staining revealed the lack of collagen fibers in the SV compared to the SL (Figure S5). The SL harbors numerous fibrocytes and collagen fibers; SV vessels are encapsulated by interdigitated marginal, intermediate, and basal cells 51. Moreover, the SV is compartmentalized from the SL by claudin-11-associated tight junctions, which form the “blood-labyrinthine barrier” 52. These harsh environments against interstitial migration may be another factor that limits neutrophil infiltration into the SV. The “blood-labyrinthine barrier” is subdivided into the “blood-endolymph barrier,” which is mainly composed of capillaries in the SV facing scala media, and the “blood-perilymph barrier,” which is mainly composed of the vessels in the SL facing the scala tympani and scala vestibule 53. The exclusive infiltration of neutrophils in the SL and perilymph spaces implies that under inflammatory conditions, the “blood-perilymph barrier” is loosened, but the “blood-endolymph barrier” remains tight for immune cell migration. The functionally isolated and distinct region in the SV leads to spatially different responses after LPS injection and likely aims to protect the organ of Corti in the cochlea, which is surrounded by the scala media, whereas the SL abutting the scala tympani properly regulates immune responses according to the stimuli.

To date, investigation of the cochlea using TPIM has been limited in explanted samples 31,54,55. To the best of our knowledge, this study is the first to evaluate intravital imaging of the lateral wall of the cochlea. To overcome the anatomic hurdles for approaching the cochlea, precise surgical procedures must be performed. Mice were alive for at least 4 h during the intravital imaging, which was the longest possible time in this experiment. The surgery did not induce lethality throughout the experiment. Although the cortical bone of the cochlea is not highly transparent, it was feasible to visualize the cellular structures of the cochlear lateral wall and analyze cell tracking. Even though the intact cortical bone limits the resolution and the depth of the image, we did not remove the cortical bone to guarantee the protection of the inner structures from outer conditions, including physical disruption, heat, and blood.

Intravital imaging allowed us to quantify and analyze crawling neutrophils, which could not be investigated in a sample obtained from euthanized mice including *ex vivo* or explanted cochlea. This method can be extended to any cell type that is tagged by a fluorescent protein. For instance, resident cochlea macrophages were visualized in the CX3CR1-GFP^+/-^ mice in our previous experiments 31,56. The structural or vascular changes would also be possible to investigate* in vivo*, considering that the results of the current study demonstrated most of the vessels in the lateral wall of the cochlea. However, our study may have a few limitations. First, GFP-positive cells in LysM-GFP^+/-^ mice were not fully identified in the cochlea. GFP-positive crawling cells might not be the only neutrophils; some monocytes might also be involved. Further studies using catchup Ly6G mice, which is a more neutrophil-specific model, could demonstrate a clearer result 57. Nevertheless, it is reasonable that crawling cells after LPS inoculation are mostly neutrophils, as LPS inoculation induces massive interstitial migration of neutrophils after the peak time in the population of the crawling cells. Second, the resolution and tissue depth of the cochlea imaging technology were limited. If it were feasible to create a bony window without damaging the SL, it might result in the improvement of the image resolution and working distance. However, it was technically challenging to remove the cortical bone without damaging the inner structure, as the cortical bone is very thin, and the cortical vessels beneath it may be disrupted. Consequently, visualizing the capillary network in the lower part of the SL was difficult compared to the SV because of the thick bone capsule, although ICAM-1 expression was different between the SV and SL. Third, the surgical procedure destroys hearing function, which may be overcome using the endoscopic approach via the EAC 58. Fourth, the inflammation induced by LPS does not correspond to the actual otitis media model. However, many previous studies have used a similar or higher amount of LPS middle ear inoculation to induce inner ear inflammation 38,59. Furthermore, inner ear inflammation induced by bacterial injection to the middle ear showed a more detrimental effect on the inner ear structure than that caused by LPS, as demonstrated by previous studies 24,60. Lastly, the detrimental effect of neutrophils was not investigated, although it is important for clinical applications. Similar to other organs, excessive infiltration of neutrophils might aggravate immune responses and tissue damage 25,26. We expect further studies to demonstrate the effect of neutrophil infiltration in the future.

In conclusion, our novel intravital imaging method of live mouse cochlea revealed that neutrophil recruitment and infiltration during LPS-induced inflammation is spatially controlled and exclusively observed in the SL but not the SV, which is likely attributed to the differential response of ICAM-1 in the cochlea.

## Supplementary Material

Supplementary figures and videos.

Supplementary video S1.

Supplementary video S2.

Supplementary video S3.

## Abbreviations

EAC: external auditory canal
SV: stria vascularis
SL: spiral ligament
TPIM: two-photon intravital microscopy
EAC: external auditory canal
DAMP: damage associated molecular pattern
LPS: lipopolysaccharide
IL: interleukin
