# Supplementary material for: Neutrophils infiltrate into the spiral ligament but not the stria vascularis in the cochlea during lipopolysaccharide-induced inflammation
Source: Theranostics. 2021 Jan 1;11(6):2522–33. doi: 10.7150/thno.49121 (PMC7806478; doi:10.7150/thno.49121)
Supplement: Supplementary file 1 — Supplementary figures and videos. [file thnov11p2522s1.pdf]

Supplementary Figures

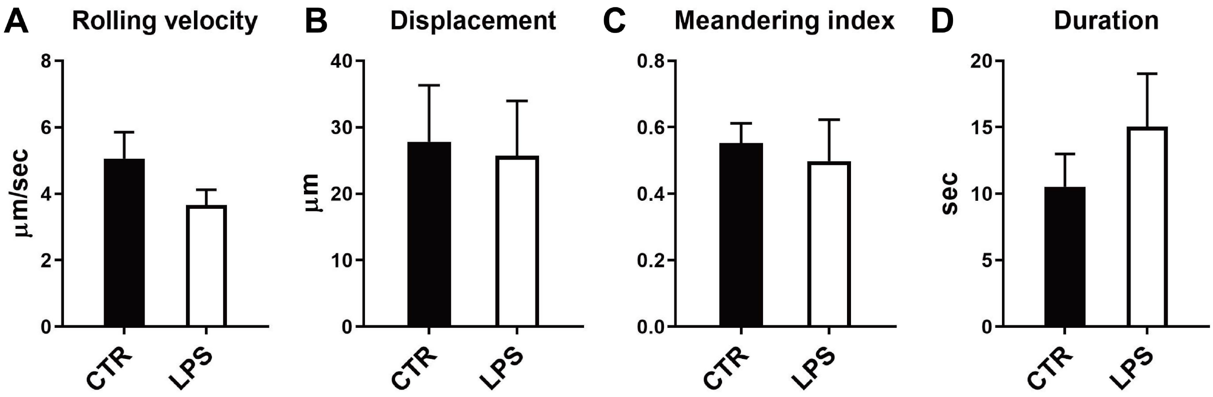

Figure S1. Dynamic characteristics of rolling neutrophils in the stria vascularis were not different between untreated and LPS-treated mice

Intravital image analysis of intravascular rolling neutrophils showed no significant differences in the rolling velocity (A), displacement (B), meandering index (C), and duration (D). The analyses included seven cells in the control group and six cells in the LPS group.

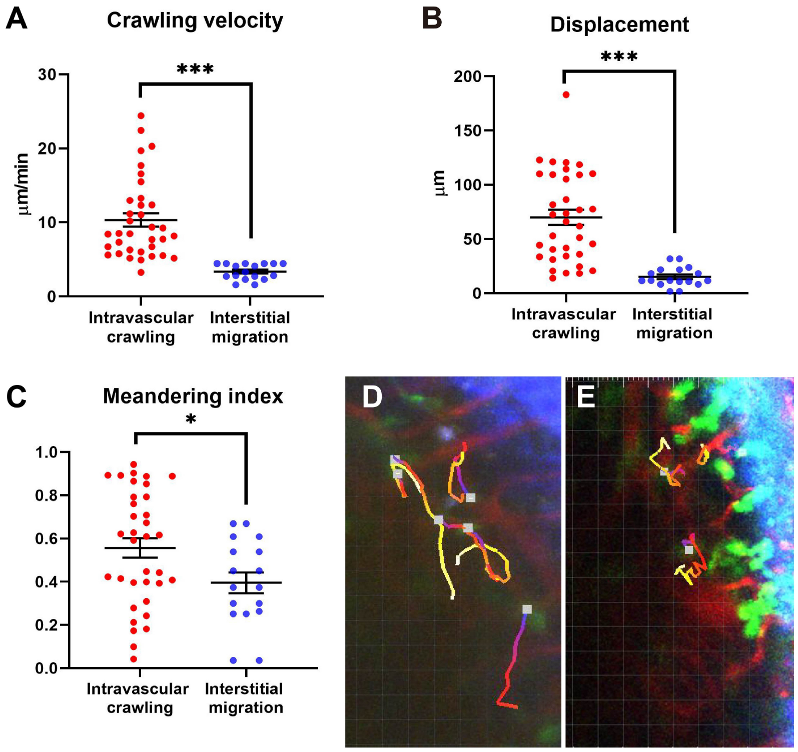

Figure S2. Intravital imaging confirmed that intravascular crawling cells have high velocity and displacement compared to interstitial migrating cells

(A-C) Intravascular crawling cells and interstitial migrating cells were compared by tracking the speed of the two groups of cells (A), tracking the displacement of the two groups of cells (B), and tracking the meandering index of the two groups of cells (C). (D-E) Tracking of the intravascular crawling cells 1 day after LPS inoculation (D) and tracking of the interstitial migrating cells 2 days after LPS inoculation (E). The cells that appeared for more than four consecutive time sequences (3 min) were selected for track analysis. The images were obtained after i.v. injection of Texas Red-dextran. The *green* region shows LysM-GFP positive cells. The *red* region shows blood vessels stained with Texas Red-dextran. *Blue* region shows the second harmonic generation of cortical bone. In (B) and (D), a large grid denotes 10 μm (* denotes P-value < 0.05, *** denotes P-value < 0.001).

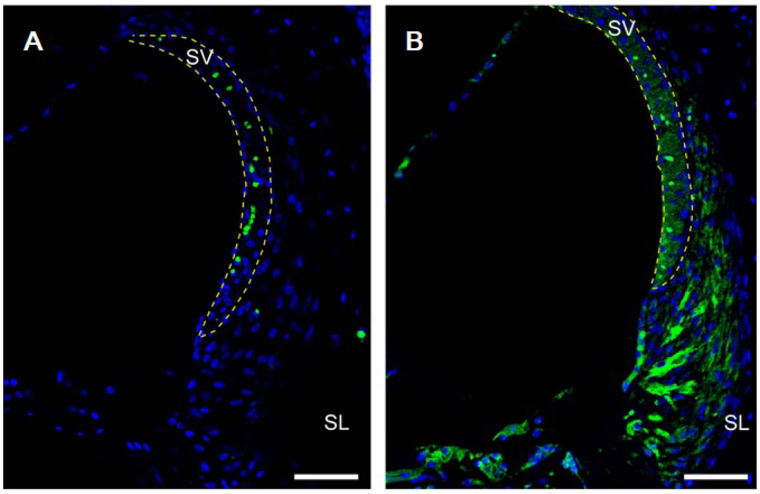

Figure S3. IL-1β levels increased in both the stria vascularis and spiral ligament 1 day after LPS injection

Immunofluorescence using IL-1β antibody was conducted in paraffin sections of untreated cochlea basal turn (A), and cochlea basal turn at 1 day after LPS injection (B). The fluorescence signal intensity increased in both the stria vascularis and spiral ligament after LPS injection, but the lower part of the spiral ligament showed the highest intensity. Notably, autofluorescence from red blood cells inside vessels is present in both the conditions. IL-1β stained *green*. *SV*, stria vascularis. *SL*, spiral ligament. Scale bar = 50 μm.

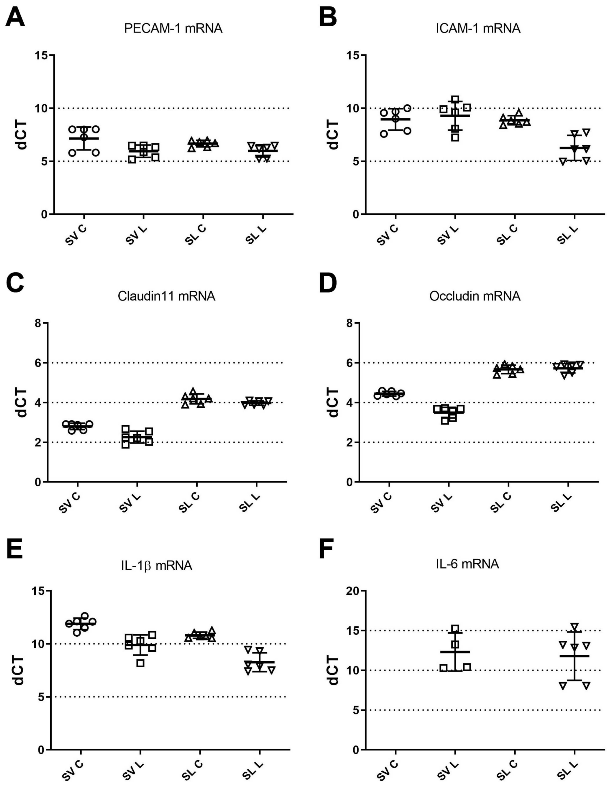

Figure S4. *PECAM-1, ICAM-1, CLDN11, OCLN, IL-1β,* and *IL-6* mRNA expression at baseline and 1 day after LPS injection differed between the stria vascularis and spiral ligament as analyzed by qPCR

(A-B) Change in the mRNA expression of *PECAM-1* and *ICAM-1*, which are genes associated with leukocyte transmigration. *ICAM-1* mRNA expression increased 1 day after LPS injection only in the spiral ligament. (C-D) Change in the mRNA expression of claudin-11 and occludin, which are genes associated with tight junctions. Both genes were highly expressed in the stria vascularis at baseline and 1 day after LPS injection. (E-F) Change in the mRNA expression of *IL-1β* and *IL-6*, which encode cytokines associated with an inflammatory reaction. Both genes were increased at 1 day after LPS injection in both the stria vascularis and spiral ligament. Notably, *IL-6* mRNA was not detected at the baseline in both the stria vascularis and spiral ligament and two LPS-injected stria vascularis samples. The dots indicate delta CT (dCT) value that is calculated by subtracting the CT value of *GAPDH* from the CT value of the target gene. *SV C*; untreated stria vascularis, *SV L*; LPS-injected stria vascularis, *SL C*; untreated spiral ligament, *SL L*; LPS-injected spiral ligament.

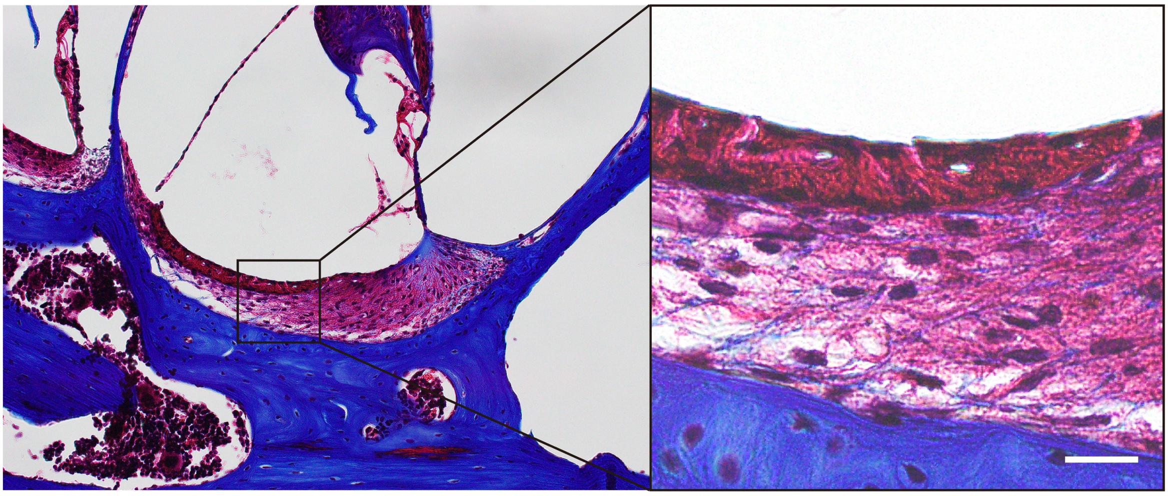

Figure S5. Spiral ligament consisted of abundant collagen fibers, but the stria vascularis did not

The untreated cochlea stained using Masson's trichrome method. The image on the right side is a magnified view of the black lined square in the left image. The collagen fibers were stained *blue*. The cytosol was stained *red*. The nucleus was stained *dark brown*. The white bar on the right image indicates 20 μm.

Supplementary Video Legends

Supplementary Video 1. Intravital imaging of the untreated control mouse cochlear lateral wall. The image obtained after i.v. Texas Red-dextran injection. The *green* region shows LysM-GFP positive cells. The *red* region shows blood vessels stained with Texas Red-dextran. The *blue* region shows the second-harmonic generation of cortical bone.

Supplementary Video 2. Intravital imaging of mouse cochlear lateral wall 1 day after middle ear inoculation with LPS. The image obtained after i.v. Texas Red-dextran injection. The *green* region shows LysM-GFP positive cells. The *red* region shows blood vessels stained with Texas Red-dextran. The *blue* region shows the second-harmonic generation of cortical bone.

Supplementary Video 3. Intravital imaging of the mouse cochlear lateral wall 2 days after LPS middle ear inoculation. The representative interstitial migrating cells were tracked with a blue line. The image obtained after i.v. Texas Red-dextran injection. *The green* region shows LysM-GFP positive cells. *The red* region shows blood vessels stained with Texas Red-dextran. *The blue* region shows the second-harmonic generation of cortical bone.
